# Th17/Treg imbalance and clinical characteristics in psoriasis patients with metabolic dysfunction-associated steatotic liver disease: a cross-sectional analysis

**DOI:** 10.3389/fimmu.2026.1958145

**Published:** 2026-10-09

**Authors:** Sai Dong, Zixuan Qiu, Zheng Zhao, Hui Bao, Xiaoyang Liu, Zhanglei Mu, Jianzhong Zhang, Houmin Li, Bo Feng, Lin Cai

**Affiliations:** 1 Department of Dermatology, Peking University People’s Hospital, Beijing, China; 2 Department of Hepatology, Peking University People’s Hospital, Peking University Hepatology Institute, Beijing, China; 3 Department of Dermatology, Peking University International Hospital, Beijing, China; 4 Health Management Center, Peking University People’s Hospital, Beijing, China

**Keywords:** BMI, hyperuricemia, insulin resistance, metabolic dysfunction-associated steatotic liver disease (MASLD), psoriasis, Th17 cells, Treg cells, vibration-controlled transient elastography (VCTE)

## Abstract

**Background:**

Psoriasis is a chronic systemic inflammatory disease that imposes a considerable burden of metabolic comorbidities. Metabolic dysfunction-associated steatotic liver disease (MASLD) is particularly common among patients with psoriasis, which may be attributed to shared metabolic and immune-inflammatory pathogenic pathways. However, evidence linking peripheral T-cell profiles to clinical phenotypes remains limited.

**Methods:**

This is a cross-sectional study. A total of 408 participants were enrolled stratified into three groups: 206 patients with plaque psoriasis, 100 subjects undergoing routine health examinations (RHE group), and 102 patients with isolated MASLD. All participants underwent vibration-controlled transient elastography (VCTE) via FibroScan®, with simultaneous acquisition of Liver Stiffness Measurement (LSM) and Controlled Attenuation Parameter (CAP). Comprehensive clinical and laboratory data were collected for further analysis. In a subset of 49 psoriasis participants (32 patients with psoriasis and MASLD and 17 patients with psoriasis alone), peripheral T-cell subpopulations were quantified using flow cytometry.

**Results:**

Psoriasis patients had a higher MASLD prevalence than participants in RHE group (65.5% vs. 46.0%, P = 0.002), with psoriasis independently associated with MASLD (adjusted OR = 2.282, 95% CI: 1.181-4.408). Body mass index (BMI), hyperuricemia, insulin resistance-related indices (triglyceride glucose index [TyG], TyG-BMI), and uric acid-to-high-density lipoprotein cholesterol ratio (UHR) were linked to concomitant MASLD in psoriasis patients. Elevated liver enzymes were associated with severe hepatic steatosis grades. An exploratory model combining BMI, TyG and hyperuricemia exhibited favorable performance for MASLD screening, with an area under the receiver operating characteristic curve (AUC) of 0.84 (95% CI, 0.78-0.89). Compared with patients with psoriasis alone, those with coexisting MASLD showed elevated circulating IL-17+ Th17 cells and an increased Th17/Treg ratio, and this immune imbalance correlated positively with the grade of hepatic steatosis.

**Conclusion:**

Psoriasis is independently associated with MASLD. Psoriasis patients with coexisting MASLD present metabolic abnormalities along with peripheral Th17/Treg immune imbalance. The exploratory model combining BMI, the TyG index and hyperuricemia shows good performance for MASLD screening. These findings highlight the importance of multidisciplinary management for patients with psoriasis.

## Introduction

Psoriasis is a chronic, immune-mediated, inflammatory skin disease with multi-organ involvement. It is often accompanied by multiple comorbidities, including psoriatic arthritis (PsA), metabolic syndrome (MetS), obesity, type 2 diabetes mellitus (T2DM), dyslipidemia, cardiovascular disease, metabolic dysfunction-associated steatotic liver disease (MASLD), etc. MASLD, as the most prevalent chronic liver disease globally, exhibits significantly higher prevalence among psoriasis populations and aggravates systemic metabolic disturbance and chronic inflammatory burden.

Several studies indicate that the prevalence of MASLD is significantly higher in psoriasis patients than in the general population (approximately 30%-50% vs. 20%-30%) (1, 2). Metabolic-associated syndrome, characterized by obesity and insulin resistance, is recognized as a shared risk factor for both psoriasis and MASLD, which may account for the mechanistic overlap between the two disorders (3–6). Additionally, psoriasis and MASLD share overlapping immunoinflammatory pathogenic pathways driven by elevated tumor necrosis factor (TNF)-α, interleukin (IL)-1, IL-6, lowered circulating adiponectin and Th17/Treg imbalance (7–10).

Although previous studies have described the epidemiological association between psoriasis and MASLD, most reports focus merely on clinical correlation or single inflammatory markers. Comprehensive profiling of circulating Th1, Th17, and Treg and their relationship with clinical and hepatic phenotypes remains scarce. Therefore, the present cross-sectional study systematically compared clinical, metabolic, and hepatic characteristics between psoriasis patients with and without MASLD. Furthermore, we detected peripheral immune indices in a subgroup to clarify alterations in Th17/Treg immune profiles. This study aimed to reveal the potential immunological mechanism underlying psoriasis-MASLD comorbidity and provide novel inflammatory biomarkers for early screening and stratified clinical management of high-risk psoriasis populations.

## Materials and methods

### Study design and ethical approval

This cross-sectional analysis was performed based on baseline data derived from a prospective study. All psoriasis patients were administered standardized treatment with regular follow-up. This study was conducted in accordance with the Declaration of Helsinki and approved by the Ethics Committee of Peking University People’s Hospital (2025PHB194-001). All enrolled participants were divided into three groups: patients with plaque psoriasis, MASLD patients without psoriasis, and subjects undergoing routine health examinations (RHE). All participants provided written informed consent prior to study enrollment.

### Sample size calculation

Sample size calculation was performed using PASS version 25.0.3 (NCSS, LLC, Kaysville, UT, USA). Based on previously reported MASLD prevalence rates of 44% in psoriasis patients and 25% in the general population (11), we designed this study with a 2:1 enrollment ratio of psoriasis patients to RHE participants and a 10% dropout allowance. With a two-sided α of 0.05 and 85% statistical power, the two-sample unequal proportion test indicated a minimum sample of 278 participants, including 185 psoriasis patients and 93 routine health-examination subjects. Given the 44% comorbidity rate of MASLD among psoriasis patients, an additional isolated MASLD group was enrolled, forming a 2:1:1 enrollment ratio among psoriasis group, RHE group, and isolated MASLD group.

### Study population and data collection

The study enrolled adult psoriasis patients from Department of Dermatology, Peking University People’s Hospital between April 2025 and February 2026. Participants in the RHE group were recruited from the hospital’s Health Management Center, and patients with isolated MASLD were enrolled from the Department of Hepatology during the same period. Drug-induced liver injury, viral hepatitis, immune-mediated liver disease, or excessive alcohol consumption (ethanol intake ≥210 g/week for men and ≥140 g/week for women) were excluded based on medication history, liver biochemical tests, viral hepatitis screening, autoimmune-related laboratory findings, and other relevant clinical information. Diagnostic criteria for MASLD includes steatotic liver disease confirmed by FibroScan® and at least one of five cardiometabolic criteria (12). All MASLD diagnoses were confirmed by hepatologists. Participants in the RHE group were frequency-matched to psoriasis group by sex, with the sex distribution observed in the psoriasis group (approximate male-to-female ratio of 6:4). Patients with isolated MASLD were frequency-matched to PsO-MASLD patients by sex (approximate male-to-female ratio of 6:4) and CAP-defined hepatic steatosis grade (approximate mild-to-moderate-to-severe distribution of 2:3:5). Detailed diagnostic, inclusion and exclusion criteria are provided in the **Supplementary Materials**.

Clinical data were collected, including demographic information (age, sex, and body mass index [BMI]), clinical profiles (history of MetS, T2DM, hypertension, and hyperuricemia, as well as alcohol consumption status, etc.), psoriasis-related indicators (Psoriasis Area and Severity Index [PASI], Body Surface Area [BSA], static Physician Global Assessment [sPGA], etc.) and laboratory parameters.

Two insulin resistance indices, namely the triglyceride-glucose (TyG) index and TyG-BMI, were calculated using the following formulas: TyG=ln [triglyceride (mg/dL)×fasting blood glucose (mg/dL)/2]; TyG-BMI = TyG×BMI (kg/m2).

### Liver assessment

Liver steatosis, quantified by the controlled attenuation parameter (CAP) and hepatic fibrosis, quantified by liver stiffness measurement (LSM), were assessed using vibration-controlled transient elastography (VCTE) with a FibroScan® device (Echosens, Paris, France). Examinations were performed after overnight fasting, with participants lying in the supine position and their right arm placed behind the head. The probe was placed over the right hepatic lobe through an intercostal space. The M or XL probe was selected according to the manufacturer’s recommendation. At least 10 valid measurements were obtained at the same site using the same probe. The median values of valid measurements were used for subsequent analyses and measurements were considered reliable when IQR/median ≤30%. All examinations were performed by trained sonographers.

The grade of steatosis was defined based on CAP value according to the center's criteria: mild steatosis (238-259dB/m), moderate steatosis (259-292dB/m), and severe steatosis (≥292 dB/m). Fibrosis status was assessed using LSM value, with significant fibrosis defined as LSM ≥8 kPa (12).

### Flow cytometry

A consecutive subset of 49 patients with psoriasis underwent immune profiling by flow cytometry. Fresh peripheral whole blood samples were collected and processed within 24 hours of collection. Flow cytometry was applied to quantify the proportions of multiple CD4^+^T cell subsets, including interferon (IFN)-γ^+^(CD3+CD4+IFN+), TNF-α^+^ (CD3+CD4+ TNF-α+), and IL-2^+^ Th1-related cells (CD3+CD4+IL-2+), IL-4^+^ Th2 cells (CD3+CD4+IL-4+), IL-17A^+^ Th17 cells (CD3+CD4+IL-17A+), and regulatory T (Treg) cells (CD3+CD4+CD25highFoxp3+).

For intracellular cytokine analysis, heparinized blood was stimulated by using Leukocyte Activation Cocktail and BD GolgiPlugTM (4 hours, 37 °C). Cells were surface-stained with anti-CD3-PerCP and anti-CD4-APC antibodies, fixed and permeabilized with the BD IntraSureTM Kit. Then cells were stained intracellularly with anti-IL-2-FITC, anti-TNF-α-PE, anti-IFN-γ-FITC, anti-IL-4-PE, and anti-IL-17A-PE antibodies (all from BD Biosciences). Detailed information on antibody clones is provided in **Supplementary Table S1**.

For Treg analysis, EDTA-anticoagulated cells were surface-stained with anti-CD3-PerCP, anti-CD4-APC and anti-CD25-PE antibodies (all from BD Biosciences). After fixation and permeabilization, cells were stained with anti-FoxP3-AF647 antibody (from BioLegend).

Samples for Treg analysis was acquired using a BD FACSLyric flow cytometer (BD Biosciences), whereas samples for Th1, Th2, and Th17 analysis were acquired using a BriCyte E6 flow cytometer (Mindray). Data were analyzed using MRFlow software (Mindray, Shenzhen, China) and BD FACSDiva software (BD Biosciences, San Jose, CA, USA).

All the lymphocyte population was identified on the basis of forward scatter (FSC) and side scatter (SSC) characteristics. Gating strategies are provided in **Supplementary Figure 1**.

### Statistical analysis

All statistical analyses were performed using IBM SPSS Statistics version 27.0 (IBM Corp., Armonk, NY, USA) or GraphPad Prism version 10.6 (GraphPad software, LLC, San Diego, CA, USA). Continuous variables were presented as mean ± standard deviation (SD) or median with interquartile range (IQR). Categorical data were shown as numbers and percentage. The student’s t-test or Mann-Whitney U test were used for quantitative comparisons according to data distribution. The chi-squared test or Fisher’s exact test was used for categorical comparisons.

Univariate logistic regression, multivariate logistic regression and multiple linear regression were used to identify independent factors associated with MASLD. Results are presented as odds ratios (OR) and 95% confidence intervals (CI). Furthermore, the area under the curve (AUC), the specificity and the sensitivity were calculated to evaluate model discriminatory performance. Spearman’s rank correlation analysis was used to evaluate the associations of Th17 cells and the Th17/Treg ratio with PASI, BSA, CAP, and CAP-stratified hepatic steatosis grades. The Benjamini-Hochberg false discovery rate (BH-FDR) procedure was applied to the between-group comparisons of immune profiles. A P-value < 0.05 was regarded as statistically significant.

## Results

### Baseline characteristics of study populations

A total of 408 participants were enrolled in this study, including 206 patients with plaque psoriasis, 100 subjects in the RHE group, and 102 patients with isolated MASLD. Age, sex, and BMI were comparable between psoriasis patients and subjects in the RHE group (**Table 1**). Similarly, no significant differences in age, sex, or hepatic steatosis grades were observed between patients with MASLD with and without psoriasis (**Table 2**).

**Table 1:** Baseline clinical characteristics of psoriasis patients and routine health-examination group.

Variables	Total PsO	RHE group	*P* value
	N=206	N=100	
Age (years)	40.62 ± 14.02	43.25 ± 10.55	0.069
Sex (male)	126 (61.2%)	58 (58.0%)	0.685
Height (cm)	169.37 ± 8.10	169.75 ± 8.12	0.700
Weight (kg)	72.06 ± 13.47	69.91 ± 12.43	0.182
BMI (kg/m2)	25.02 ± 3.77	24.18 ± 3.50	0.062
Underweight (<18.5)	10 (4.9%)	5 (5.0%)	1.000
Normal weight (18.5-24)	69 (33.5%)	45 (45.0%)	0.068
Overweight (24–28)	83 (40.3%)	39 (39.0%)	0.927
Obesity (≥28)	44 (21.4%)	11 (11.0%)	0.040*
Liver assessment			
LSM (kPa)	4.60 (3.82-5.50)	4.20 (3.50-5.30)	0.020*
CAP (dB/m)	261.74 ± 58.28	237.75 ± 57.26	<0.001***
MASLD	135 (65.5%)	46 (46.0%)	0.002**
Mild steatosis	28 (13.6%)	12 (12.0%)	0.836
Moderate steatosis	44 (21.4%)	16 (16.0%)	0.340
Severe steatosis	63 (30.6%)	18 (18.0%)	0.028*
Comorbidities			
Hypertension	18 (8.7%)	9 (9.0%)	1.000
Hyperlipidemia	58 (28.2%)	34 (34.0%)	0.361
Cardiovascular disease	4 (1.9%)	1 (1.0%)	1.000
Diabetes mellitus	13 (6.3%)	3 (3.0%)	0.344
Hyperuricemia	69 (33.5%)	24 (24.0%)	0.118
MetS	25 (12.1%)	4 (4.0%)	0.038*
Blood routine			
Neutrophil (10^9^/L)	4.04 (3.28-5.08)	3.33 (2.68-4.29)	<0.001***
Lymphocyte (10^9^/L)	2.08 (1.75-2.57)	1.90 (1.60-2.26)	0.002**
Monocyte (10^9^/L)	0.36 (0.30-0.45)	0.34 (0.26-0.39)	0.004**
Eosinophil (10^9^/L)	0.11 (0.06-0.16)	0.11 (0.08-0.19)	0.177
Basophil (10^9^/L)	0.03 (0.02-0.04)	0.03 (0.02-0.04)	0.116
Platelet (10^9^/L)	249.82 ± 63.86	248.28 ± 50.64	0.833
Blood Biochemistry			
Uric acid (umol/L)	353.00 (288.00, 426.75)	347.57 ± 91.26	0.303
Blood glucose (mmol/L)	4.90 (4.65-5.47)	4.75 (4.41-5.09)	0.001**
TC (mmol/L)	4.86 ± 0.93	4.96 ± 1.07	0.375
TG (mmol/L)	1.30 (0.98-1.94)	1.13 (0.82-1.75)	0.017*
HDL (mmol/L)	1.12 (1.02-1.33)	1.29 (1.09-1.56)	<0.001***
LDL (mmol/L)	2.91 (2.44, 3.54)	2.98 (2.60-3.75)	0.118
ALT (U/L)	22.00 (15.00-35.00)	18.00 (13.00-26.25)	0.012**
AST (U/L)	22.00 (18.00-27.75)	20.00 (17.00-24.00)	0.051
γ-GT (U/L)	22.50 (15.00-38.75)	23.50 (15.00-36.00)	0.664
FIB-4	0.69 (0.51-1.06)	0.92 ± 0.38	0.007**
Insulin resistance indices			
TyG	8.58 (8.20, 8.99)	8.41 ± 0.58	0.002**
TyG-BMI	214.14 (188.91, 243.31)	204.24 ± 37.24	0.017*
Inflammatory parameters			
NLR	1.95 (1.51-2.43)	1.78 (1.32-2.32)	0.070
SII	465.98 (346.01-606.72)	419.88 (319.44-568.27)	0.160
UHR	301.49 (216.33, 405.74)	276.61 ± 106.23	0.004**

Hyperuricemia refers to serum uric acid level >420mmol/L in men and >360 mmol/L in women.

TC, total cholesterol; HDL, high-density lipoprotein cholesteroPLR, platelet-to-lymphocyte ratio; SII, systemic immune-inflammation index; UHR, uric acid-to-HDL ratio.

* P<0.05, **P<0.01, ***P<0.001

**Table 2:** Clinical characteristics of patients with MASLD with or without psoriasis. .

Variables	PsO-MASLD	MASLD	*P* value
	N=135	N=102	
Age (years)	41.69 ± 14.31	42.70 ± 11.02	0.541
Sex (male)	86 (63.7%)	68 (66.7%)	0.737
Height (cm)	169.46 ± 8.23	170.15 ± 7.47	0.510
Weight (kg)	75.83 ± 13.17	79.92 ± 14.23	0.023*
BMI (kg/m^2^)	26.31 ± 3.53	27.54 ± 4.26	0.016*
Underweight (<18.5)	2 (1.5%)	0 (0.0%)	0.508
Normal weight (18.5-24)	28 (20.7%)	18 (17.6%)	0.667
Overweight (24-28)	64 (47.4%)	45 (44.1%)	0.710
Obesity (≥28)	41 (30.4%)	39 (38.2%)	0.259
Liver assessment			
LSM (kPa)	5.00 (4.25, 5.80)	5.40 (4.50, 6.67)	0.011*
CAP (dB/m)	289.00 (266.00, 326.00)	301.32 ± 40.68	0.278
MASLD			
Mild steatosis	28 (20.7%)	16 (15.7%)	0.411
Moderate steatosis	44 (32.6%)	31 (30.4%)	0.826
Severe steatosis	63 (46.7%)	55 (53.9%)	0.330
Comorbidities			
Hypertension	13 (9.6%)	22 (21.6%)	0.017*
Hyperlipidemia	46 (34.1%)	53 (52.0%)	0.009**
Cardiovascular disease	2 (1.5%)	4 (3.9%)	0.407
Diabetes mellitus	10 (7.4%)	13 (12.7%)	0.249
Hyperuricemia	62 (45.9%)	48 (47.1%)	0.967
MetS	23 (17.0%)	21 (20.6%)	0.598
Laboratory parameters			
Blood routine			
Neutrophil (10^9^/L)	4.10 (3.31, 5.12)	3.58 (3.05, 4.62)	0.021*
Lymphocyte (10^9^/L)	2.13 (1.81, 2.68)	2.20 ± 0.68	0.751
Monocyte (10^9^/L)	0.38 (0.32, 0.46)	0.40 (0.29, 0.50)	0.963
Eosinophil (10^9^/L)	0.12 (0.07, 0.19)	0.13 (0.07, 0.21)	0.637
Basophil (10^9^/L)	0.03 (0.02, 0.04)	0.03 (0.02, 0.04)	0.295
Platelet (10^9^/L)	257.13 ± 65.61	251.70 ± 56.18	0.503
Blood Biochemistry			
Uric acid (umol/L)	386.18 ± 103.90	393.40 ± 91.38	0.577
Blood glucose (mmol/L)	4.96 (4.69, 5.70)	5.11 (4.69, 5.63)	0.464
TC (mmol/L)	4.98 ± 0.91	4.88 ± 1.13	0.501
TG (mmol/L)	1.54 (1.12, 2.39)	1.62 (1.23, 2.46)	0.206
HDL (mmol/L)	1.09 (0.97, 1.25)	1.15 ± 0.22	0.317
LDL (mmol/L)	3.09 ± 0.70	3.07 ± 0.82	0.836
ALT (U/L)	25.00 (17.00, 45.50)	42.00 (26.25, 79.75)	<0.001***
AST (U/L)	23.00 (18.00, 30.00)	28.50 (23.00, 42.00)	<0.001***
γ-GT (U/L)	27.00 (18.00, 44.00)	42.00 (26.00, 64.75)	<0.001***
FIB-4	0.67 (0.50, 1.07)	0.78 (0.56, 1.18)	0.073
Insulin resistance indices			
TyG	8.83 ± 0.62	8.92 ± 0.63	0.263
TyG-BMI	230.45 (204.18–261.68)	237.38 (211.73, 275.43)	0.054*
Inflammatory parameters			
NLR	1.93 (1.47, 2.29)	1.70 (1.38, 2.22)	0.164
SII	456.70 (346.10, 619.63)	425.42 (329.61, 585.55)	0.253
UHR	345.76 (258.24, 440.68)	358.16 ± 122.53	0.951

Hyperuricemia refers to serum uric acid level >420mmol/L in men and >360 mmol/L in women.

TC, total cholesterol; HDL, high-density lipoprotein cholesterol; LDL, low-density lipoprotein cholesterol; FIB-4, Fibrosis-4 index; NLR, neutrophil-to-lymphocyte ratio; SII, systemic immune-inflammation index; UHR, uric acid-to-HDL ratio.

* P<0.05, **P<0.01, ***P<0.001

### Comparison of clinical characteristics of psoriasis patients, RHE participants, and isolated MASLD patients

Of the enrolled psoriasis patients, 135 had comorbid MASLD. Compared to subjects in the RHE group, psoriasis patients exhibited higher prevalence of overall MASLD (65.5% vs. 46.0%, P = 0.002) and severe steatosis (30.6% vs. 18.0%, P = 0.028), along with elevated LSM (4.60[3.82-5.50] vs. 4.20[3.50-5.30] kPa, P = 0.020) and CAP values (261.74 ± 58.28 vs. 237.75 ± 57.26 dB/m, P<0.001) (**Table 1**). Multivariable logistic regression confirmed that psoriasis constitutes an independent correlate of MASLD (adjusted OR = 2.282, 95% CI: 1.181-4.408, P = 0.014) (**Supplementary Table S2**). Subgroup stratification by psoriasis severity revealed no significant differences in the distribution of hepatic steatosis grades among patients with MASLD (**Table 3**).

**Table 3:** Distribution of steatosis grade, fibrosis grade, and liver enzymes status across mild, moderate, and severe psoriasis groups.

Variables	Mild PsO	Moderate PsO	Severe PsO	*P* value
Total	87	70	49	
Steatosis				0.416
CAP-found no steatosis	27 (31.03%)	25 (35.71%)	19 (38.78%)	
Mild steatosis	11 (12.64%)	14 (20.00%)	3 (6.12%)	
Moderate steatosis	19 (21.84%)	13 (18.57%)	11 (22.45%)	
Severe steatosis	30 (34.48%)	18 (25.71%)	16 (32.65%)	
Fibrosis				
Fibrosis	6 (6.90%)	2 (2.86%)	2 (4.08%)	0.551
Liver enzyme levels				
Elevated ALT	17 (19.54%)	7 (10.00%)	10 (20.41%)	0.195
Elevated AST	9 (10.34%)	2 (2.86%)	5 (10.20%)	0.168
Elevated γ-GT	13 (14.94%)	6 (8.57%)	3 (6.12%)	0.218

The grade of psoriasis was determined by PASI: 1) mild psoriasis PASI<5; 2) moderate psoriasis PASI ≥5, <10; 3) severe psoriasis PASI ≥10.

Comparisons between isolated MASLD and PsO-MASLD patients revealed that the isolated MASLD group had a higher BMI (27.54 ± 4.26 vs. 26.31 ± 3.53 kg/m², P = 0.016), higher ratio of hypertension and hyperlipidemia. In addition, patients with isolated MASLD exhibited elevated LSM values (5.40 [4.50, 6.67] vs. 5.00 [4.25, 5.80] kPa, P = 0.011) as well as increased serum levels of liver enzymes (alanine aminotransferase [ALT], aspartate aminotransferase [AST] and gamma-glutamyl transferase [γ-GT]) (**Table 2**).

Compared with psoriasis patients without MASLD, PsO-MASLD patients had higher body weight (75.83 ± 13.17 vs. 64.88 ± 10.92 kg, P<0.001) and BMI (26.31 ± 3.53 vs. 22.58 ± 2.93 kg/m2, P<0.001). In addition, the PsO-MASLD group had a higher hyperuricemia prevalence and elevated uric acid, total cholesterol (TC), triglyceride (TG), liver enzyme levels, TyG, TyG-BMI, and uric acid-to-high-density lipoprotein cholesterol ratio (UHR) (345.76 [258.24, 440.68] vs. 244.96 [182.58, 329.29], P<0.001) (**Table 4**).

**Table 4:** Clinical characteristics of patients with psoriasis with or without MASLD.

Variables	PsO-only	PsO-MASLD	*P* value
	N=71	N=135	
Age (years)	38.38 ± 13.68	41.69 ± 14.31	0.132
Sex (Male)	40 (56.3%)	86 (63.7%)	0.379
Smoking	3 (4.2%)	14 (10.4%)	0.209
Drinking	5 (7.0%)	30 (22.2%)	0.010*
BSA	5.50 (2.75, 10.50)	5.00 (2.75, 8.75)	0.303
<3%	18 (25.4%)	34 (25.2%)	1.000
3-10%	31 (43.7%)	72 (53.3%)	0.241
≥10%	22 (31.0%)	29 (21.5%)	0.183
PASI	6.60 (3.80, 10.10)	5.50 (3.60, 9.00)	0.239
<5	27 (38.0%)	60 (44.4%)	0.461
5-10	25 (35.2%)	45 (33.3%)	0.908
≥10	19 (26.8%)	30 (22.2%)	0.579
BMl (kg/m2)	22.58 ± 2.93	26.31 ± 3.53	<0.001***
Underweight (<18.5)	8 (11.3%)	2 (1.5%)	0.004**
Normal weight (18.5-24)	41 (57.7%)	28 (20.7%)	<0.001***
Overweight (24-28)	19 (26.8%)	64 (47.4%)	0.006**
Obesity (≥28)	3 (4.2%)	41 (30.4%)	<0.001***
Comorbidities			
Hypertension	5 (7.0%)	13 (9.6%)	0.715
Hyperlipemia	12 (16.9%)	46 (34.1%)	0.015*
Cardiovascular disease	2 (2.8%)	2 (1.5%)	0.609
Diabetes mellitus	3 (4.2%)	10 (7.4%)	0.549
Hyperuricemia	7 (9.9%)	62 (45.9%)	<0.001***
MetS	2 (2.8%)	23 (17.0%)	0.006*
PsA	10 (14.1%)	21 (15.6%)	0.940
Blood routine			
Neutrophil (10^9^/L)	4.01 (3.26, 4.90)	4.10 (3.31, 5.12)	0.584
Lymphocyte (10^9^/L)	2.00 (1.58, 2.31)	2.13 (1.81, 2.68)	0.028*
Monocyte (10^9^/L)	0.33 (0.27, 0.43)	0.38 (0.32, 0.46)	0.004**
Eosinophil (10^9^/L)	0.08 (0.05, 0.12)	0.12 (0.07, 0.19)	<0.001***
Basophil (10^9^/L)	0.02 (0.02, 0.04)	0.03 (0.02, 0.04)	0.007**
Platelet (10^9^/L)	235.90 ± 58.34	257.13 ± 65.61	0.023*
Blood Biochemistry			
Uric acid (umol/L)	314.30 ± 70.52	386.18 ± 103.90	<0.001***
Blood glucose (mmol/L)	4.80 (4.62, 5.11)	4.96 (4.69, 5.70)	0.014*
TC (mmol/L)	4.60 (3.98, 5.05)	4.98 ± 0.91	0.005*
TG (mmol/L)	1.04 (0.81, 1.37)	1.54 (1.12, 2.39)	<0.001***
HDL (mmol/L)	1.26 (1.07, 1.44)	1.09 (0.97, 1.25)	<0.001***
LDL (mmol/L)	2.61 (2.26, 3.12)	3.09 ± 0.70	0.002**
ALT (U/L)	17.00 (13.00, 24.00)	25.00 (17.00, 45.50)	<0.001***
AST (U/L)	20.00 (17.00, 25.00)	23.00 (18.00, 30.00)	0.007**
γ-GT (U/L)	16.00 (13.50, 26.00)	27.00 (18.00, 44.00)	<0.001***
FIB-4	0.73 (0.54, 1.00)	0.67 (0.50, 1.07)	0.646
Insulin resistance indices			
TyG	8.35 ± 0.50	8.83 ± 0.62	<0.001***
TyG-BMI	189.01 ± 29.94	230.45 (204.18–261.68)	<0.001***
Inflammatory parameters			
NLR	2.17 ± 0.82	1.93 (1.47, 2.29)	0.081
SII	469.01 (345.47, 603.43)	456.70 (346.10, 619.63)	0.880
UHR	244.96 (182.58, 329.29)	345.76 (258.24, 440.68)	<0.001***

Hyperuricemia refers to serum uric acid level >420mmol/L in men and >360 mmol/L in women.

TC, total cholesterol; HDL, high-density lipoprotein cholesterol; LDL, low-density lipoprotein cholesterol; FIB-4, Fibrosis-4 index; NLR, neutrophil-to-lymphocyte ratio; SII, systemic immune-inflammation index; UHR, uric acid-to-HDL ratio.

* P<0.05, **P<0.01, ***P<0.001

### Associated factors of MASLD in psoriasis patients

Regression analyses were performed to identify factors associated with MASLD among patients with psoriasis. BMI, serum uric acid, hyperuricemia, TC, TG, liver enzymes, TyG, TyG-BMI and UHR were significantly associated with MASLD. After adjustment for confounding covariates, all these biomarkers remained independent correlates of MASLD in separate multivariate logistic and linear regression models (**Table 5**, **Supplementary Table S3**). Additionally, elevated liver enzymes (ALT, AST and γ-GT) were independently associated with severe hepatic steatosis (**Supplementary Table S4**).

**Table 5:** Crude and multivariable-adjusted logistic regression analyses of factors associated with MASLD in psoriasis patients.

Exposures	Model 1	Model 2 ^a #^	Model 3 ^b #^
	Crude OR (95% CI)	Adjusted OR (95% CI)	Adjusted OR (95% CI)
BMI (per 1kg/m_2_ increase)	1.424 (1.269-1.597)	1.466 (1.292-1.663)	1.461(1.286-1.661)
BMI <24 kg/m_2_	Ref.	Ref.	Ref.
Overweight	5.502 (2.775-10.910)	5.645 (2.735-11.650)	5.304 (2.546-11.051)
Obesity	22.322 (6.349-78.478)	24.285 (6.705-87.954)	24.093 (6.571-88.339)
Uric acid (per 1umol/L increase)	1.009 (1.005-1.012)	1.012 (1.006-1.017)	1.012 (1.006-1.017)
Hyperuricemia	7.765 (3.317-18.177)	5.828 (2.272-14.949)	6.260 (2.375-16.503)
TC (per 1mmol/L increase)	1.525 (1.093-2.127)	1.718 (1.151-2.565)	1.616 (1.078-2.422)
TG (per 1mmol/L increase)	2.846 (1.697-4.771)	1.913 (1.083-3.379)	1.766 (1.007-3.098)
ALT (per 1U/L increase)	1.044 (1.021-1.068)	1.034 (1.009-1.059)	1.034 (1.009-1.059)
AST (per 1U/L increase)	1.052 (1.013-1.093)	1.049 (1.004-1.096)	1.052 (1.005-1.101)
γ-GT (per 1U/L increase)	1.052 (1.026-1.078)	1.035 (1.007-1.065)	1.032 (1.003-1.062)
TyG (per SD increase)	2.695 (1.810-4.013)	1.992 (1.253-3.165)	1.876 (1.172-3.003)
Q1	Ref.	Ref.	Ref.
Q2	2.563 (1.279-5.136)	1.795 (0.801-4.020)	1.659 (0.730-3.769)
Q3	6.463 (2.903-14.389)	3.526 (1.397-8.898)	3.095 (1.204-7.960)
TyG-BMI (per SD increase)	4.745 (2.907-7.744)	5.608 (3.207-9.806)	5.397 (3.076-9.468)
Q1	Ref.	Ref.	Ref.
Q2	3.680 (1.816-7.459)	3.776 (1.771-8.050)	3.357 (1.551-7.268)
Q3	22.528 (8.010-63.359)	24.787 (8.335-73.715)	22.621 (7.539-67.876)
UHR (per SD increase)	2.852 (1.845-4.408)	3.697 (1.904-7.182)	3.808 (1.925-7.529)
Q1	Ref.	Ref.	Ref.
Q2	1.989 (1.004-3.938)	2.658 (0.984-7.180)	2.630 (0.948-7.300)
Q3	7.708 (3.310-17.952)	12.194 (3.498-42.502)	13.458 (3.675-49.286)

^a^Adjusted for age, sex and BMI.

^b^Adjusted for age, sex, BMI, smoking, and drinking.

# BMI was not included as an adjustment variable in the models evaluating BMI or TyG-BMI.

Due to significant intercorrelation among TyG, TyG-BMI, and UHR, each variable was analyzed in a separate multivariate regression model to mitigate multicollinearity. TyG, TyG-BMI, and UHR were additionally stratified into tertiles (Q1-Q3) in ascending order.

Furthermore, receiver operating characteristic (ROC) curve analysis was performed. The combined model incorporating BMI, TyG and hyperuricemia exhibited good discriminatory ability for detecting MASLD in psoriasis patients (**Figure 1**), with an AUC of 0.84 (95% CI: 0.78-0.89), a sensitivity of 0.68 (95% CI: 0.62-0.74), a specificity of 0.81 (95% CI: 0.72-0.90), and F1-score of 0.76 (95% CI: 0.72-0.80). These findings indicated that the combined model had a good discriminatory ability for identifying MASLD in psoriasis patients.

**Figure 1:**
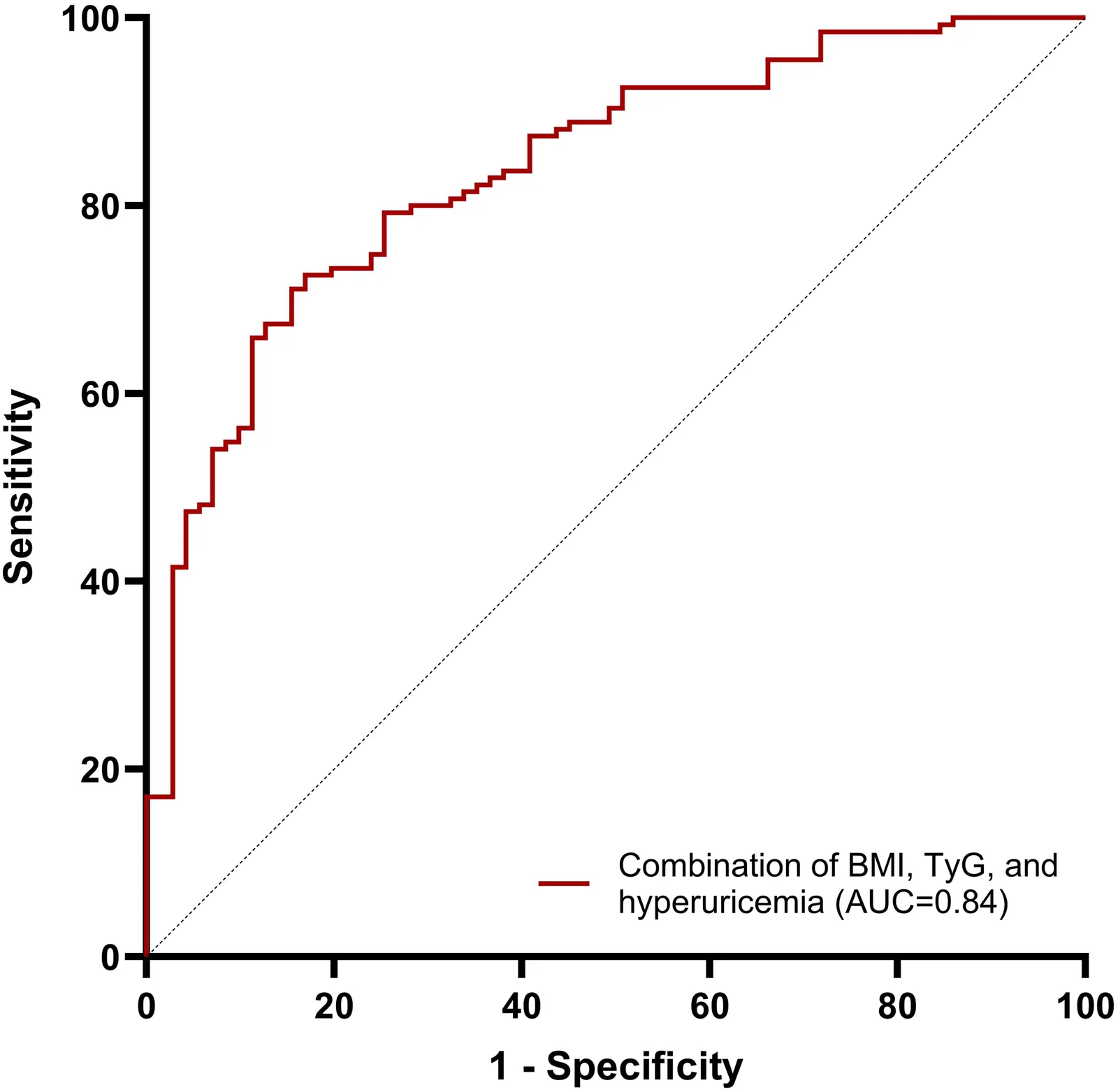
Receiver operating characteristic (ROC) curve for the combined model in identifying MASLD in patients with psoriasis.

### Comparison of immune profiles between psoriasis patients with and without MASLD

Th1, Th2, Th17 and Treg cells subsets were assessed in a consecutive subset of 49 participants, including 32 patients with PsO-MASLD and 17 patients with psoriasis alone. The Th17/Treg ratio was subsequently calculated. Baseline characteristics comparing the immune subgroup with the overall psoriasis cohort, as well as comparisons between the two clinical groups within the immune subgroup, were presented in **Supplementary Tables S5**– **Supplementary Tables S7**.

Compared with patients with psoriasis alone, patients with PsO-MASLD had significantly higher proportions of IL-17+ Th17 cells (BH-FDR q=0.0105) and elevated Th17/Treg ratio (BH-FDR q=0.0021) (**Supplementary Table S8**; **Figures 2E,G**). No significant differences were observed in the proportions of IFN-γ^+^ Th1 cells, TNF-α^+^ Th1 cells, IL-2+Th1 cells, IL-4+ Th2 cells, or Treg cells between the two groups (**Figures 2A–D, F**).

**Figure 2:**
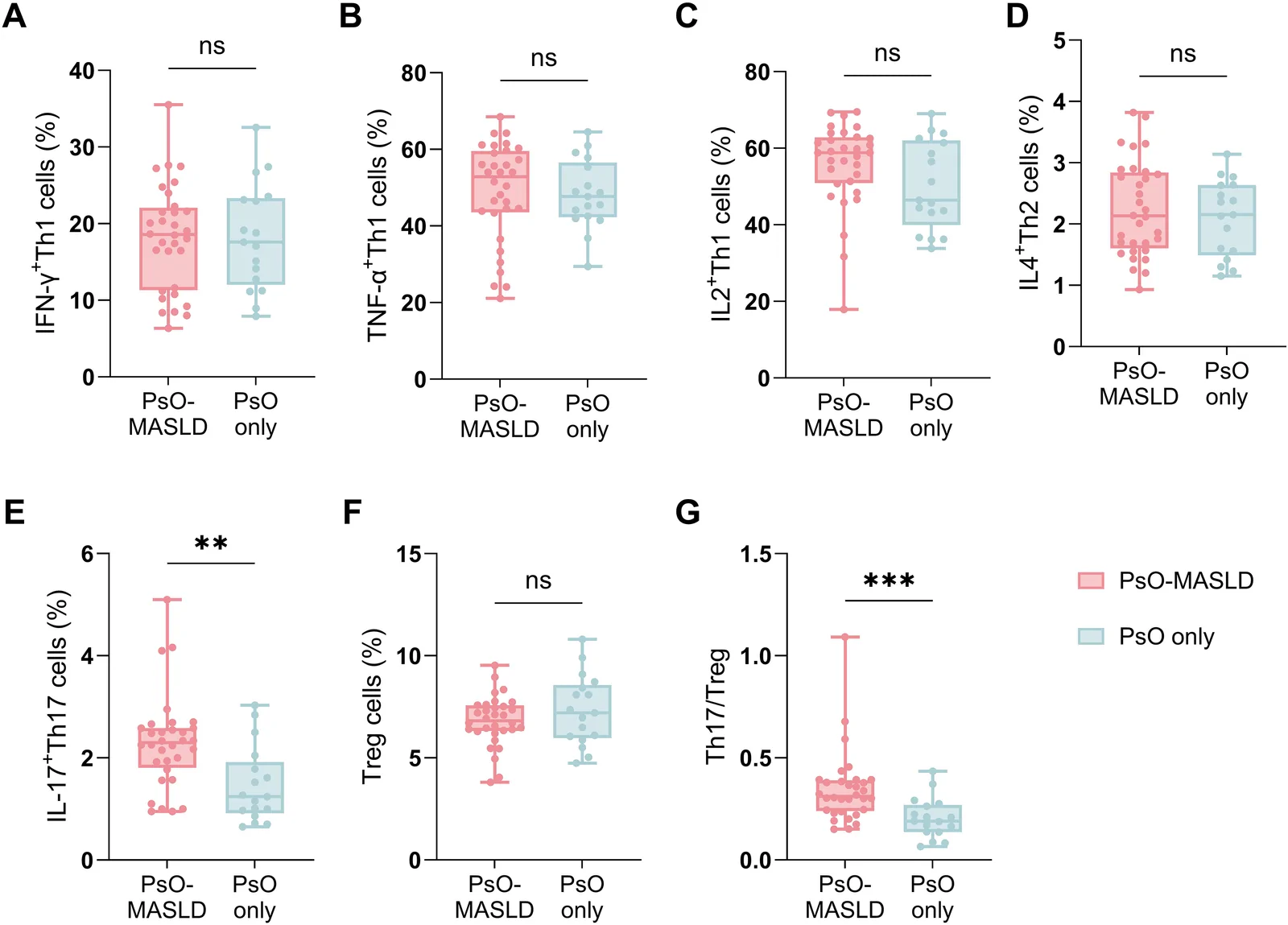
Cell subpopulations in psoriasis patients with and without MASLD. Boxplots displaying the frequencies of IFN-γ+Th1 **(A)**, TNF-α+Th1 **(B)**, IL-2+Th1 **(C)**, IL-4+Th2 **(D)**, IL-17+Th17 **(E)**, Treg **(F)** cells, and Th17/Treg ratio **(G)**. Th1, Th2, Th17 and Treg cells subsets were assessed in 49 participants, including 32 patients with PsO-MASLD and 17 patients with PsO alone, and the Th17/Treg ratio was calculated accordingly. Boxplots show median, IQR and whiskers represent the minimum and maximum values. The data were analyzed by the Mann-Whitney U test. *P< 0.05, **P < 0.01, ***P < 0.001.

Spearman rank correlation analyses were performed to explore whether Th17-related immune indices correlated with the severity of psoriasis or hepatic steatosis grades (**Table 6**). Th17/Treg ratio was positively correlated with CAP values (ρ=0.375, BH-FDR q=0.032) and CAP-stratified hepatic steatosis grades (ρ=0.405, BH-FDR q = 0.032). In contrast, neither Th17 cell proportions nor the Th17/Treg ratio was significantly correlated with psoriasis severity (PASI or BSA, all P>0.05). Additional multivariable analyses, including a multivariable linear regression analysis with CAP value as a continuous variable and a binary logistic regression analysis with MASLD as the outcome were performed. The Th17/Treg ratio remained significantly associated with MASLD in the binary logistic regression analysis (adjusted OR = 6.751, 95% CI: 1.026-44.404, P = 0.047) (**Table 7**), but not in the multivariable linear regression model (**Supplementary Table S9**). The discrepancy between the two models may be partly explained by the limited sample size and the different outcome definitions.

**Table 6:** Correlations of Th17-related immune measures with psoriasis severity and hepatic steatosis.

Immune measure	Outcome	Spearman ρ	*P* value	BH-FDR *q* value
Th17 cells	PASI	-0.242	0.093	0.149
	BSA	-0.214	0.140	0.187
	CAP	0.286	0.046	0.092
	CAP-stratified steatosis grades	0.322	0.024	0.064
Th17/Treg ratio	PASI	-0.109	0.454	0.519
	BSA	-0.086	0.555	0.555
	CAP	0.375	0.008	0.032
	CAP-stratified steatosis grades	0.405	0.004	0.032

**Table 7:** Binary logistic regression analyses of the associations between immune parameters and MASLD.

Exposures	Model 1		Model 2 ^a^		Model 3 ^b^	
	Crude OR (95% CI)	*P*	Adjusted OR (95% CI)	*P*	Adjusted OR (95% CI)	*P*
Th17 (per 1% increase)	3.969 (1.521-10.362)	0.005**	3.495 (1.121-10.900)	0.031*	3.086 (0.968-9.838)	0.057
Treg (per 1% increase)	0.769 (0.498-1.185)	0.234	0.759 (0.426-1.353)	0.350	0.804 (0.446-1.450)	0.469
Th17/Treg (per SD increase)	7.314 (1.924-27.800)	0.003**	7.759 (1.284-46.909)	0.026*	6.751 (1.026-44.404)	0.047*

^a^Adjusted for age, sex and BMI.

^b^Adjusted for age, sex, BMI and TyG.

## Discussion

Consistent with previous epidemiological evidence, psoriasis as an independent risk factor for MASLD. In this study, the frequency of MASLD was significantly higher in psoriasis patients than in RHE group, which is consistent with previous reports (1, 2, 13, 14). Prior studies have linked higher PASI scores to concurrent MASLD in psoriasis patients (1), while Miele et al. found no such association (2). In this study, no significant correlation was detected between MASLD and psoriasis severity. Notably, the PsO-MASLD group achieved comparable hepatic steatotic severity to isolated MASLD patients despite lower BMI, implying that psoriasis-related chronic inflammation partly contributes to their liver steatosis. Hence, this difference indicates overlapping metabolic mechanisms but distinct pathogenic pathways between the two conditions.

Patients with isolated MASLD had higher body weight and BMI, consistent with the well-established role of obesity in MASLD. Adipose tissue is a vital endocrine organ that secretes inflammatory mediators, including IL-1, IL-6 and TNF-α, regulating immunity and metabolic homeostasis. White adipose tissue-derived mediators trigger persistent low-grade chronic inflammation in obese populations (15). Obesity acts as an independent risk factor for both psoriasis and MASLD (16–18). Therefore, targeting obesity may be a potential therapy for such patients.

This study found that both TyG and TyG-BMI were significantly associated with MASLD in psoriasis patients. As reliable emerging biomarkers for insulin resistance, TyG and TyG-BMI are closely correlated with metabolic comorbidities including T2DM and MASLD, particularly in overweight or obese individuals with psoriasis, and have been widely used in clinical research (19). Consistent with our findings, Zhong et al. also reported a positive association of TyG and TyG-BMI with MASLD prevalence in psoriasis patients (20). Moreover, our study further validates the good discriminatory performance.

FibroScan®-derived parameters were employed in the present study. While liver biopsy remains the diagnostic gold standard for MASLD, non-invasive biomarkers and examination are increasingly used to assess therapeutic efficacy in clinical trials of liver diseases (21). FibroScan® is more sensitive than abdominal ultrasonography for detecting hepatic steatosis (22). A meta-analysis reported that the prevalence of MASLD among psoriasis patients was 63% when assessed using transient elastography, compared with 43% when assessed using abdominal ultrasonography (23).

This study also found that liver enzyme levels were significantly elevated in patients with PsO-MASLD compared with those with psoriasis alone. Regression analysis revealed that elevated liver enzymes were independently associated with severe steatosis, indicative of aggravated liver injury. Notably, the predominant elevations of ALT and AST observed in our cohort reflects a hepatocellular injury pattern, consistent with previous findings that elevated ALT is independently associated with hepatic steatosis, whereas γ-GT serves as an independent correlator of significant liver fibrosis (24, 25). The observed increase in γ-GT may be attributed to its role in glutathione metabolism (26). As the most abundant intracellular antioxidant, glutathione is heavily consumed under oxidative stress conditions prevalent in both psoriasis and MASLD. Consistent with this, a nationwide cohort study has identified elevated γ-GT as a marker associated with psoriasis risk (27).

This study showed that elevated serum uric acid levels and hyperuricemia were positively correlated with PsO-MASLD. Kwon et al. proposed elevated serum uric acid in psoriasis patients to accelerated epidermal cell turnover (28), though this mechanism remains controversial. One study reported a hyperuricemia prevalence of approximately 20.7% among patients with psoriatic arthritis (29). As one kind of alarmin, urate crystals activate the innate immune response and promote the production of pro-inflammatory cytokines such as TNF-α, IL-1α, IL-8 and IL-6, which play a critical role in the pathogenesis of psoriasis (30, 31).

Previous studies reported that serum uric acid levels in patients with psoriasis correlated with BMI and blood lipids, but showed no significant association with age, disease duration or PASI (32, 33). Hyperuricemia is also closely linked to MetS. A retrospective study identified hyperuricemia as an independent correlate of MetS (34). Uric acid aggravates insulin resistance by inhibiting the biological activity of nitric oxide. Inflammatory responses and uric acid-induced changes in the oxidative status of adipocytes also promote the development and progression of MetS (35). Accumulating evidence have demonstrated the association between uric acid and MASLD (36, 37). These results further validate the strong correlation across psoriasis, hyperuricemia, and MASLD.

This study revealed that UHR as an independent correlate of MASLD. This indicator integrates two core metabolic markers, serum uric acid and high-density lipoprotein cholesterol (HDL). The role of uric acid in MASLD has been discussed above, while reduced HDL level is one of the typical characteristic manifestations of MetS. Therefore, the UHR may serve as a more comprehensive and sensitive indicator for assessing MASLD. Studies have shown that elevated UHR is independently associated with the risk of MASLD in patients with T2DM and obese children in China (38, 39). Further, increased UHR is correlated with a higher risk of cardiovascular disease and all-cause mortality risk in MASLD patients (40, 41). Collectively, these findings indicate that UHR, as a novel metabolic biomarker, acts as an important discriminatory indicator for MASLD.

Th17/Treg imbalance may contribute to psoriasis progression through inflammatory pathways. The present study identified elevated frequencies of Th17 cells and an increased Th17/Treg ratio among patients with psoriasis complicated by MASLD. Both Th17 cell proportions and the Th17/Treg ratio were positively correlated with continuous CAP values and CAP-stratified hepatic steatosis grades. Similarly, Supruniuk et al. reported increased Th17 cells and decreased Treg cells frequencies in patients with MASLD relative to healthy controls, and they proposed that Th17/Treg ratio may reflect MASLD severity (10). In our cohort, this immune dysregulation showed no significant correlation with psoriasis severity. This discrepancy may reflect immune compartmentalization between the peripheral circulation and cutaneous lesions. Circulating Th17-cell frequencies do not consistently track clinical disease improvement, and pathogenic Th17 subsets or lesional resident Th17 cells may be more closely related to cutaneous disease activity than total circulating IL-17+ Th17 cells.

Th17/Treg imbalance may also drive MASLD progression through inflammatory pathways shared with psoriasis (42, 43). In high-fat-diet mouse models, upregulated hepatic URI expression triggers hepatocellular DNA damage and promotes hepatic Th17 cells recruitment together with IL-17A secretion. Sustained IL-17A signaling activates hepatic macrophages and hepatic stellate cells, thereby inducing hepatocyte injury and accelerating the progression of fatty liver disease (44). Interruption of the IL-17 signaling axis alongside restoration of the Treg/Th17 balance alleviates hepatic steatosis in murine models and holds therapeutic potential for mitigating hepatic inflammation (44, 45). Several case reports have indicated that biologics targeting the IL-17/IL-23 axis can improve MASLD in limited cases (46, 47). Collectively, these preclinical and clinical findings suggest that therapeutic targeting of Th17 cells represents a potential intervention strategy against MASLD.

Our findings recommend routine MASLD screening for psoriatic patients complicated with obesity, insulin resistance and hyperuricemia. Those with abnormal liver enzymes require referral to hepatology and subsequent multidisciplinary team (MDT) management. For patients with concurrent psoriasis and MASLD, lifestyle interventions including regular exercise, dietary restriction and targeted MetS therapy are essential to attenuate disease initiation and progression. Previous studies have reported that combination therapy with Ixekizumab and Tirzepatide (a dual GIP/GLP-1 receptor agonist) achieved superior PASI 75 and PASI 100 improvement at week 36, accompanied by greater weight reduction, which further contributed to the improvement of MASLD (48, 49). However, few studies have comprehensively assessed how antipsoriatic treatments, especially when administered long-term, alongside lifestyle interventions affect MASLD progression, indicating a critical gap in relevant therapeutic research.

This study has several limitations. First, the study is cross-sectional analysis, therefore, causal relationships between associated factors and MASLD cannot be inferred. Second, the sample size of the immune subgroup is relatively small and restricted exclusively to patients with psoriasis, which limits the generalizability of immune-related findings. Third, although several potential confounders were incorporated into the multivariable models, residual confounding from unmeasured factors, including lipid-lowering medication use, may still be present. In addition, only peripheral blood immune parameters were assessed in this study, which may not fully reflect immune changes in skin or liver tissues. Finally, the exploratory regression model and immune findings have not undergone external validation in an independent cohort. Further large-sample, prospective studies and mechanistic experiments are required to further clarify the precise regulatory mechanisms of Th17/Treg dysregulation in psoriasis-MASLD comorbidity.

## Conclusion

The present study delineates characterizes the clinical, metabolic, and immune profiles of patients with concurrent psoriasis and MASLD, further supporting an underlying inflammatory linkage between the two disorders. Psoriasis was identified as an independently associated with MASLD. BMI, uric acid, hyperuricemia, TyG, TyG-BMI, and UHR are potential correlates of MASLD, whereas liver enzymes (ALT, AST and γ-GT) are associated with higher steatosis grades. A model combining BMI, TyG, and hyperuricemia showed favorable discriminatory performance for MASLD in psoriasis patients. Regular screening for MASLD among psoriasis patients complicated with obesity, insulin resistance or hyperuricemia may be recommended. Collectively, these observations underscore the necessity of multidisciplinary management of psoriatic patients and provide evidence for future investigations into liver-related impacts of long-term psoriasis medications.

## Data Availability

The raw data supporting the conclusions of this article will be made available by the authors, without undue reservation.
