# Supplementary material for: Th17/Treg imbalance and clinical characteristics in psoriasis patients with metabolic dysfunction-associated steatotic liver disease: a cross-sectional analysis
Source: Front Immunol. 2026 Oct 9;17:1958145. doi: 10.3389/fimmu.2026.1958145 (PMC13690623; doi:10.3389/fimmu.2026.1958145)
Supplement: Supplementary file 1 [file Supplementaryfile1.docx]

***Supplementary Material***

**Diagnostic criteria for MASLD, inclusion and exclusion criteria**

1. Diagnostic criteria for MASLD includes steatotic liver disease confirmed by FibroScan^®^ and at least one of five cardiometabolic criteria:
(a) BMI ≥24.0 kg/m^2^, waist circumference ≥90 cm in men or ≥85 cm in women, or excess body fat;
(b) fasting plasma glucose ≥6.1 mmol/L, 2-h post-load plasma glucose ≥7.8 mmol/L, HbA1c ≥5.7%, a history of type 2 diabetes mellitus, or a HOMA-IR ≥2.5;
(c) triglycerides ≥1.70 mmol/L or lipid-lowering treatment;
(d) HDL ≤1.0 mmol/L in men or ≤1.3 mmol/L in women or lipid-lowering treatment;
(e) blood pressure ≥130/85 mmHg or antihypertensive treatment.

Drug-induced liver injury, viral hepatitis, immune-mediated liver disease, or excessive alcohol consumption (ethanol intake ≥210 g/week for men and ≥140 g/week for women) were excluded.

All MASLD diagnoses were confirmed by hepatologists after excluding alternative causes of liver disease based on medication history, liver biochemical tests, viral hepatitis screening, autoimmune-related laboratory findings, and other relevant clinical information.

2. Eligible participants were enrolled based on the following criteria:
(a) Plaque psoriasis patients were clinically or histopathologically diagnosed by dermatologists.
(b) Participants in the RHE group were frequency-matched to psoriasis group by sex, with the sex distribution observed in the psoriasis group (approximate male-to-female ratio of 6:4). Patients with isolated MASLD were frequency-matched to PsO-MASLD patients by sex (approximate male-to-female ratio of 6:4) and CAP-defined hepatic steatosis grade (approximate mild-to-moderate-to-severe distribution of 2:3:5).
(c) Excluded psoriasis phenotypes included guttate, pustular, erythrodermic, and drug-induced psoriasis.
(d) No psoriasis patients received biologics within 3 months, systemic immunomodulatory agents within 1 month, and phototherapy or topical treatment within 2 weeks prior to baseline data collection.
(e) Individuals with pregnancy, malignancy, other immune-mediated diseases (e.g. inflammatory bowel disease, rheumatoid arthritis), drug-induced liver injury, viral hepatitis, immune-mediated liver disease, or alcoholic liver disease were excluded.
(f) Patients with severe infections were excluded.
(g) Patients with unstable, progressive, or uncontrolled illnesses (renal, hematological, gastrointestinal, endocrine, pulmonary, cardiac, neurological, psychiatric, or cerebral diseases) that might affect clinical evaluation or study participation were excluded.
(h) Patients with incomplete or missing data were excluded.

**Supplementary Table 1** Monoclonal antibodies used in flow cytometric analysis

|  | **Antigen** | **Clone** | **Fluorochrome** | **Vendor** |
| --- | --- | --- | --- | --- |
| Th1/2/17 cells | CD3 | SK7 | PerCP | BD |
|  | CD4 | SK3 | APC | BD |
|  | IFN-γ | 25723.11 | FITC | BD |
|  | TNF-α | MAb11 | PE | BD |
|  | IL-2 | 5344.111 | FITC | BD |
|  | IL-4 | 3010.211 | PE | BD |
|  | IL-17A | N49-653 | PE | BD |
| Treg | CD3 | SK7 | PerCP | BD |
|  | CD4 | SK3 | FITC | BD |
|  | CD25 | 2A3 | PE | BD |
|  | Foxp3 | 206D | AF647 | BioLegend |

**Supplementary Table 2** Logistic regression analyses of the association between psoriasis and MASLD

| **Variables** | **Model 1** | | **Model 2^a^** | | **Model 3^b^** | |
| --- | --- | --- | --- | --- | --- | --- |
|  | Crude OR (95% CI) | *P* | Adjusted OR (95% CI) | *P* | Adjusted OR (95% CI) | *P* |
| **Psoriasis** | 2.232 (1.371-3.633) | 0.001 | 2.183 (1.237–3.854) | 0.007** | 2.282 (1.181–4.408) | 0.014* |
| **Age** | — | — | 1.005 (0.984–1.027) | 0.620 | 1.008 (0.983–1.034) | 0.528 |
| **Sex (Male)** | — | — | 0.673 (0.373–1.216) | 0.189 | 0.264 (0.119–0.588) | 0.001** |
| **BMI**  (per 1kg/m^2^ increase) | — | — | 1.493 (1.339–1.663) | <0.001*** | 1.428 (1.255–1.623) | <0.001*** |
| **ALT**  (per 1U/L increase) | — | — | — | — | 1.016 (0.976–1.058) | 0.428 |
| **AST**  (per 1U/L increase) | — | — | — | — | 0.996 (0.936–1.061) | 0.903 |
| **γ-GT**  (per 1U/L increase) | — | — | — | — | 1.013 (0.990–1.037) | 0.261 |
| **HDL**  (per 1mmol/L increase) | — | — | — | — | 0.677 (0.263–1.742) | 0.419 |
| **LDL**  (per 1mmol/L increase) | — | — | — | — | 1.515 (0.967–2.373) | 0.069 |
| **Uric acid**  (per 1μmol/L increase) | — | — | — | — | 1.007 (1.003–1.012) | 0.002** |
| **TyG**  (per SD increase) | — | — | — | — | 1.168 (0.770–1.774) | 0.465 |
| **SII**  (per SD increase) | — | — | — | — | 1.089 (0.810–1.463) | 0.572 |

*^a^*Adjusted for age, sex, BMI;

*^b^*Adjusted for age, sex, BMI, ALT, AST, γ-GT, HDL, LDL, uric acid, TyG, SII.

**Supplementary Table 3** Multiple linear regression between factors associated with CAP value in psoriasis patients

| **Exposures** | **Model 1** | | **Model 2**^a#^ | |
| --- | --- | --- | --- | --- |
|  | 0β (95% CI) | *P* | 1β (95% CI) | *P* |
| **BMI**  (per 1kg/m^2^ increase) | 7.547 (5.686-9.408) | <0.001*** | 7.490(5.529-9.451) | <0.001*** |
| **Uric acid**  (per 1μmol/L increase) | 0.214 (0.139-0.290） | <0.001*** | 0.181 (0.097-0.265） | <0.001*** |
| **TC**  (per 1mmol/L increase) | 13.222 (4.748-21.696) | 0.002** | 11.797 (4.259-19.336) | 0.002** |
| **TG**  (per 1mmol/L increase) | 10.158 (5.303-15.013) | <0.001*** | 5.201 (0.419-9.984) | 0.033* |
| **ALT**  (per 1U/L increase) | 0.983 (0.700-1.266) | <0.001*** | 0.776 (0.504-1.048) | <0.001*** |
| **AST**  (per 1U/L increase) | 0.337 (0.107-0.568) | 0.004** | 0.276 (0.074-0.477) | 0.008** |
| **γ-GT**  (per 1U/L increase) | 0.591 (0.386-0.796) | <0.001*** | 0.391 (0.190-0.592) | <0.001*** |
| **TyG**  (per SD increase) | 25.532 (18.300-32.765) | <0.001*** | 16.475 (8.790-24.159) | <0.001*** |
| **TyG-BMI**  (per SD increase) | 31.427 (24.652-38.203) | <0.001*** | 31.611 (24.423-38.800) | <0.001*** |
| **UHR**  (per SD increase) | 20.824 (13.309-28.338) | <0.001*** | 16.141 (7.026-25.256) | <0.001*** |

^a^Adjusted for age, sex, BMI, smoking, and drinking.

^#^BMI was not included as an adjustment variable in the models evaluating BMI or TyG-BMI.

Each variable was entered into a separate multivariate regression model to mitigate multicollinearity between correlated biomarkers.

**Supplementary Table 4** Multivariable logistic regression and subgroup analysis between liver enzymes and severe steatosis in psoriasis patients

| Subgroup | **Total** | **Sex** | Male | Female | **BMI (kg/m^2^)** | <24 | ≥24 |
| --- | --- | --- | --- | --- | --- | --- | --- |
| **ALT** | 1.070  (1.043-1.097) |  | 1.077  (1.043-1.113) | 1.064  (1.019-1.111) |  | 1.088  (1.03-1.150) | 1.074  (1.041-1.109) |
| P value | <0.001*** |  | <0.001*** | 0.005** |  | 0.003** | <0.001*** |
| P for interaction |  | 0.881 |  |  | 0.974 |  |  |
| **AST** | 1.127  (1.074-1.181) |  | 1.051  (1.074-1.234) | 1.103  (1.033-1.179) |  | 1.179  (1.065-1.304) | 1.137  (1.069-1.210) |
| P value | <0.001*** |  | <0.001*** | 0.003** |  | 0.001** | <0.001*** |
| P for interaction |  | 0.293 |  |  | 0.871 |  |  |
| **γ-GT** | 1.038  (1.016-1.061) |  | 1.030  (1.006-1.054) | 1.064  (1.016-1.115) |  | 1.142  (1.044-1.249) | 1.037  (1.016-1.059) |
| P value | <0.001*** |  | 0.014* | 0.008** |  | 0.004** | <0.001*** |
| P for interaction |  | 0.366 |  |  | 0.242 |  |  |

Adjusted for age, sex, BMI, smoking, and drinking.

**Supplementary Table 5** Clinical characteristics comparison between the immune subgroup and overall psoriasis group

| Variables | **Total PsO** | **Immune subgroup** | ***P* value** |
| --- | --- | --- | --- |
|  | N=206 | N=49 |  |
| Age (years) | 40.62±14.02 | 40.67±12.39 | 0.981 |
| Sex (male) | 126(61.2%) | 24(49.0%) | 0.163 |
| Height (cm) | 169.37±8.10 | 169.41±8.97 | 0.978 |
| Weight (kg) | 72.06±13.47 | 71.59±17.63 | 0.864 |
| **BMI (kg/m^2^)** | 25.02±3.77 | 24.74±4.55 | 0.647 |
| **Liver assessment** |  |  |  |
| LSM (kPa) | 4.60 (3.82, 5.50) | 4.60 (3.90, 5.40) | 0.750 |
| CAP (dB/m) | 261.74±58.28 | 260.98±59.83 | 0.935 |
| Mild steatosis | 28(13.6%) | 8(16.3%) | 0.790 |
| Moderate steatosis | 44(21.4%) | 11(22.4%) | 1.000 |
| Severe steatosis | 63(30.6%) | 13(26.5%) | 0.701 |
| **BSA** | 5.00 (2.62, 9.50) | 5.00 (2.00, 10.50) | 0.990 |
| **PASI** | 6.00 (3.60, 9.60) | 7.00 (3.60, 10.20) | 0.431 |
| **sPGA** | 3.00 (2.00, 3.00) | 3.00 (2.00, 4.00) | 0.834 |
| **Comorbidities** |  |  |  |
| Hypertension | 18(8.7%) | 4(8.2%) | 1.000 |
| Hyperlipemia | 58(28.2%) | 14(28.6%) | 1.000 |
| Cardiovascular disease | 4(1.9%) | 0(0.0%) | 1.000 |
| Diabetes mellitus | 13(6.3%) | 1(2.0%) | 0.318 |
| Hyperuricemia | 69(33.5%) | 9(18.4%) | 0.058 |
| PsA | 31(15.0%) | 13(26.5%) | 0.089 |
| **Previous systemic treatments** |  |  |  |
| Naive | 171(83.0%) | 37(75.5%) | 0.312 |
| Conventional oral medications | 16(7.8%) | 7(14.3%) | 0.167 |
| Biologics | 24(11.7%) | 7(14.3%) | 0.792 |

**Supplementary Table 6** Clinical characteristics comparison between the immune subgroup and overall psoriasis group

| Variables | **PsO-MASLD in total PsO** | **PsO-MASLD immune subgroup** | ***P* value** | **PsO only in total PsO** | **PsO only immune subgroup** | ***P* value** |
| --- | --- | --- | --- | --- | --- | --- |
|  | N=135 | N=32 |  | N=71 | N=17 |  |
| Age (years) | 41.69±14.31 | 42.19±13.41 | 0.858 | 38.59±13.31 | 37.82±9.95 | 0.824 |
| Sex (male) | 86(63.7%) | 19(59.4%) | 0.687 | 40(56.3%) | 5(29.4%) | 0.060 |
| Height (cm) | 169.46±8.23 | 169.97±9.02 | 0.759 | 169.20±7.91 | 168.35±9.05 | 0.702 |
| Weight (kg) | 75.83±13.17 | 76.91±18.42 | 0.702 | 64.88±10.92 | 61.59±10.60 | 0.265 |
| **BMI (kg/m^2^)** | 26.31±3.53 | 26.35±4.37 | 0.954 | 22.58±2.93 | 21.70±3.15 | 0.277 |
| **Liver assessment** |  |  |  |  |  |  |
| LSM (kPa) | 5.00 (4.25, 5.80) | 4.60 (3.88, 5.22) | 0.108 | 3.90 (3.50, 4.85) | 4.60 (3.90, 5.80) | 0.161 |
| CAP (dB/m) | 289.00  (266.00, 326.00) | 285.50  (259.75, 323.00) | 0.810 | 206.00  (177.00, 224.50) | 209.00  (176.00, 228.00) | 0.597 |
| **BSA** | 5.00 (2.75, 8.75) | 4.25 (2.00, 10.88) | 0.966 | 5.50 (2.75, 10.50) | 5.00 (2.50, 10.50) | 0.958 |
| **PASI** | 5.50 (3.60, 9.00) | 7.20 (3.60, 10.20) | 0.405 | 6.60 (3.80, 10.10) | 7.00 (4.40, 10.75) | 0.934 |
| **sPGA** | 3.00 (2.00, 3.00) | 3.00 (2.00, 4.00) | 0.746 | 3.00 (2.00, 4.00) | 3.00 (2.00, 4.00) | 0.929 |
| **Comorbidities** |  |  |  |  |  |  |
| Hypertension | 13(9.6%) | 2(6.2%) | 0.738 | 5(7.0%) | 2(11.8%) | 0.616 |
| Hyperlipemia | 46(34.1%) | 12(37.5%) | 0.837 | 12(16.9%) | 2(11.8%) | 1.000 |
| Cardiovascular disease | 2(1.5%) | 0(0.0%) | 1.000 | 2(2.8%) | 0(0.0%) | 1.000 |
| Diabetes mellitus | 10(7.4%) | 1(3.1%) | 0.693 | 3(4.2%) | 0(0.0%) | 1.000 |
| Hyperuricemia | 62(45.9%) | 9(28.1%) | 0.076 | 7(9.9%) | 0(0.0%) | 0.338 |
| PsA | 21(15.6%) | 10(31.2%) | 0.073 | 10(14.1%) | 3(17.6%) | 0.710 |
| **Previous systemic treatments** |  |  |  |  |  |  |
| Naive | 114(84.4%) | 25(78.1%) | 0.431 | 57(80.3%) | 12(70.6%) | 0.511 |
| Conventional oral medications | 10(7.4%) | 4(12.5%) | 0.475 | 6(8.5%) | 3(17.6%) | 0.367 |
| Biologics | 15(11.1%) | 5(15.6%) | 0.544 | 9(12.7%) | 2(11.8%) | 1.000 |

**Supplementary Table 7** Clinical characteristics comparison between two clinical groups within the immune subgroup

| Variables | **PsO-MASLD immune subgroup** | **PsO only immune subgroup** | ***P* value** |
| --- | --- | --- | --- |
|  | N=32 | N=17 |  |
| Age (years) | 42.19±13.41 | 37.82±9.95 | 0.245 |
| Sex (male) | 19(59.4%) | 5(29.4%) | 0.072 |
| Height (cm) | 169.97±9.02 | 168.35±9.05 | 0.554 |
| Weight (kg) | 76.91±18.42 | 61.59±10.60 | 0.003 |
| **BMI (kg/m^2^)** | 26.35±4.37 | 21.70±3.15 | <0.001 |
| **Liver assessment** |  |  |  |
| LSM (kPa) | 4.60 (3.88, 5.22) | 4.60 (3.90, 5.80) | 0.975 |
| CAP (dB/m) | 294.00±40.21 | 198.82±36.35 | <0.001 |
| **BSA** | 4.25 (2.00, 10.88) | 5.00 (2.50, 10.50) | 0.793 |
| **PASI** | 7.20 (3.60, 10.20) | 7.00 (4.80, 9.60) | 0.966 |
| **sPGA** | 3.00 (2.00, 4.00) | 3.00 (2.00, 4.00) | 0.471 |
| **Comorbidities** |  |  |  |
| Hypertension | 2(6.2%) | 2(11.8%) | 0.602 |
| Hyperlipemia | 12(37.5%) | 2(11.8%) | 0.096 |
| Cardiovascular disease | 0(0.0%) | 0(0.0%) | - |
| Diabetes mellitus | 1 (3.1%) | 0(0.0%) | 1.000 |
| Hyperuricemia | 9 (28.1%) | 2(11.8%) | 0.019 |
| PsA | 10(31.2%) | 3(17.6%) | 0.498 |
| **Previous systemic treatments** |  |  |  |
| Naive | 25(78.1%) | 12(70.6%) | 0.729 |
| Conventional oral medications | 4(12.5%) | 3(17.6%) | 0.681 |
| Biologics | 5(15.6%) | 2(11.8%) | 1.000 |

**Supplementary Table 8** Immune profile within the immune subgroup

| Variables | **PsO-MASLD immune subgroup** | **PsO only immune subgroup** | ***P* value** | **BH-FDR *q* value** |
| --- | --- | --- | --- | --- |
|  | N=32 | N=17 |  |  |
| **TNF-α^+^Th1 cells (%)** | 52.80 (43.80, 59.19) | 47.70 (42.58, 55.35) | 0.536 | 0.6253 |
| **IFN-γ^+^Th1 cells (%)** | 18.58 (11.51, 21.77) | 17.59 (12.75, 23.10) | 0.992 | 0.9920 |
| **IL-2^+^Th1 cells (%)** | 58.81 (51.20, 62.67) | 46.41 (43.23, 61.44) | 0.068 | 0.1587 |
| **IL-4^+^Th2 cells (%)** | 2.13 (1.66, 2.82) | 2.15 (1.56, 2.63) | 0.536 | 0.6253 |
| **IL-17^+^Th17 cells (%)** | 2.29 (1.88, 2.58) | 1.24 (0.97, 1.78) | 0.003 | 0.0105 |
| **Treg cells (%)** | 6.82 (6.34, 7.54) | 7.21 (6.06, 8.43) | 0.432 | 0.6253 |
| **Th17/Treg ratio** | 0.31 (0.24, 0.39) | 0.19 (0.14, 0.26) | <0.001 | 0.0021 |

**Supplementary Table 9** Multivariable linear regression analyses of the associations between immune parameters and CAP

| **Exposures** | **Model 1** | | **Model 2^a^** | | **Model 3^b^** | |
| --- | --- | --- | --- | --- | --- | --- |
|  | 0β (95% CI) | *P* | 1β (95% CI) | *P* | 2β (95% CI) | *P* |
| **Th17**  (per 1% increase) | 16.558 (-1.458-34.575) | 0.071 | 4.795 (-11.415 - 21.005) | 0.554 | 2.893 (-12.779-18.565) | 0.712 |
| **Treg**  (per 1% increase) | -12.066 (-23.859- -0.273) | 0.045 | -9.065 (-18.971- 0.840) | 0.072 | -8.277 (-17.826-1.271) | 0.088 |
| **Th17/Treg**  (per SD increase) | 23.130 (6.938-39.322) | 0.006 | 8.725 (-7.880-25.330) | 0.295 | 7.818 (-8.138-23.774)) | 0.329 |

^a^Adjusted for age, sex and BMI.

^b^Adjusted for age, sex, BMI and TyG.
